# Effect of the Formation of Hydrophilic and Hydrophobic–Hydrophilic Associates on the Behavior of Copolymers of N-Vinylpyrrolidone with Methyl Acrylate in Aqueous Solutions

**DOI:** 10.3390/polym16050584

**Published:** 2024-02-21

**Authors:** Ramazan Shaikhutdinov, Grigoriy Mun, Eldar Kopishev, Akhat Bakirov, Sherniyaz Kabdushev, Saltanat Baipakbaeva, Ibragim Suleimenov

**Affiliations:** 1 National Engineering Academy of the Republic of Kazakhstan, Almaty 050010, Kazakhstan; ramossha123@list.ru (R.S.); sherniyaz.kabdushev.hw@gmail.com (S.K.); saltanat.baipakbayeva@gmail.com (S.B.); 2 Department of Chemistry & Technology of Organic Materials, Polymers and Natural Compounds, Faculty of Chemistry and Chemical Technology, Al Farabi Kazakh National University, Almaty 050040, Kazakhstan; 3 Department of Chemistry, Faculty of Natural Sciences, L.N. Gumilyov Eurasian National University, Astana 010000, Kazakhstan; kopishev_eye@enu.kz; 4 Department of General and Inorganic Chemistry, Faculty of Natural Sciences, Bukhara State University, Bukhara 705018, Uzbekistan; 5 Department of Telecommunication Engineering, Institute of Communications and Space Engineering, Gumarbek Daukeev Almaty University of Power Engineering and Communications, Almaty 050040, Kazakhstan; a.bakirov@aues.kz

**Keywords:** interpolymer complexes, hydrophilic interpolymer associates, hydrophobic interactions, phase transitions, unstable meshes, thermosensitive polymers

## Abstract

It has been shown that there exist conditions under which thermosensitive copolymers of N-vinylpyrrolidone with methyl acrylate form hydrophobic–hydrophilic associations, which are unstable dynamic meshes, the bonds in which are continuously broken and created again, and the nature of the formation of such meshes depends significantly on the proportion of the hydrophobic component in the copolymer. It is shown that the interaction of the above copolymers with polyacrylic acid results in the formation of not only classical interpolymer complexes, but also hydrophilic interpolymer associates, which also represent unstable networks existing in a dynamic mode. In such meshes, the molecules of the above copolymers serve as a kind of cross-agent connecting the polyacid molecules. There are also conditions under which such meshes acquire a complex structure, since unstable bonds between macromolecular tangles of both the same and different types take part in their formation. It is shown that the transition from the formation of interpolymer complexes to the formation of hydrophilic interpolymer associates can occur, among other things, due to changes in the acidity or concentration of low-molecular salt in solution.

## 1. Introduction

Phase transitions in solutions of thermosensitive polymers have been studied for a long time [1,2,3,4]. This issue is not only of academic [3,5,6,7,8,9], but also of direct applied interest. In particular, the question of using polymers undergoing phase transitions to create drug delivery systems has been repeatedly raised in the literature [10,11,12,13,14,15]. It was also proposed to use phase transitions, which are expressed in in the form of sharp solution turbidity, for the creation of information display systems [16,17,18,19,20,21]. Currently, this issue is being actualized in connection with attempts to achieve optical neural networks with controlled characteristics of optical elements [22,23,24,25].

Further, the question of creating computing systems on non-traditional element bases, including chemical ones, is currently being raised [26,27,28]. In particular, in [29,30,31,32,33,34,35] the question of using stimulus-sensitive polymers to create information-processing systems on a quasi-biological basis was discussed. For the creation of such systems, it is essential that phase transitions in the systems of the considered type are accompanied by hysteresis phenomena [36,37,38,39,40,41]. The existence of a hysteresis loop means that there are conditions under which the system under consideration can be in two (or more) different states with the same set of thermodynamic parameters. At least, this allows us to speak about the possibility of recording information in the systems of the considered type by means of changing the controlling thermodynamic parameters according to a given law, which creates prerequisites for the attainment of macromolecular systems that execute one or another predetermined program. Obviously, the application of such approaches requires the deepest possible understanding of the nature of phase transitions that occur in solutions of thermosensitive polymers.

Traditionally, such transitions have been considered in terms of the tangle–globule transition [42,43,44]. Thus, the consideration of the phase transition was actually reduced to the consideration of the processes occurring in an isolated macromolecule. However, the results obtained in [45,46] show that this approach does not fully reflect the real processes occurring in polymer solutions during phase transitions.

Namely, it was shown back in [45] that a polymer solution containing a nonionic and an ionogenic component can have a rather complex structure. The formation of nonionic bonds of any type between functional groups of different macromolecules leads to the formation of a dynamic mesh in solution. Its main feature is that the bonds stabilizing it are continuously broken and reappear. As emphasized in the cited work, such a mesh actually occupies an intermediate position between true solutions, hydrogels and classical interpolymer complexes (IPC).

In [45], it was also shown that the formation of such a grid can most significantly affect the nature of the phase transition under study, which follows, among other things, from the most general qualitative reasoning.

Indeed, those interactions (e.g., hydrophilic) that lead to the formation of a globule (i.e., to the formation of an insoluble component) can play the opposite role. Specifically, they can enhance the observed solubility of the polymer when such interactions occur between functional links belonging to different macromolecular tangles, and also when macromolecules of only one type are present in solution [46]. In this case, the macromolecule is retained in solution precisely because of those interactions that would seem to favor the loss of solubility. Thus, there is every reason to assert that the analysis of the mechanism of phase transitions in solutions of thermosensitive polymers cannot be reduced to the consideration of classical tangle–globule transitions, although, of course, they cannot play more than an essential role.

This issue also has a pronounced applied aspect. Namely, a quite unexpected application of the systems of the considered type can be found in aromatherapy [47], which is becoming increasingly important as a means of correcting the psychophysiological state of society. As is known, phase transitions in solutions of thermosensitive polymers and their complementary hydrogels occur due to a shift in the hydrophobic–hydrophilic balance with temperature variations [48,49], i.e., such substances are known to be capable of hydrophobic interactions, including those with essential aromatic oils. Consequently, the controlled phase transition is able to provide controlled separation of essential oils from the solution, which allows us to obtain the corresponding systems of psychophysiological correction, operating in automatic mode, including the approach reflected in [50], enabling installation of the appropriate program on the user’s smartphone.

The aim of this work is to investigate the influence of hydrophobic–hydrophilic associates (HHAs) on phase transitions in solutions of copolymers of N-vinylpyrrolidone with methyl acrylate (NVP-MA), as well as on the formation of their complexes with polyacrylic acid (PAA). In particular, it is proven that the complex formation between the considered copolymers and PAA is influenced not only by the formation of hydrophilic interpolymer associates (HIAs) studied in [45], but also by the formation of HHAs, the existence of which was proven in [46].

We emphasize that research on the nature of both HIAs and HHAs was initiated by our research group in 2013 [45]. The very term “hydrophilic interpolymer associate” was proposed by us in this work. Other works performed in this direction are unknown to us.

## 2. Materials

*N-vinylpyrrolidone (NVP)* with 97% of the main product content, from Sigma-Aldrich (USA), was purified by double vacuum distillation (T_кип_ = 487–488 K, n_D_^20^ = 1.5120).

*Methylacrylate (MA)* containing 99% of the main product, manufactured by Aldrich (USA), was purified by water jet distillation (T_кип_ = 353 K, n_D_^20^ = 0.955).

*Pyrene* as a fluorescent label, from Sigma-Aldrich (UK), was used without further purification.

*2,2′-azobisisobutyronitrile (AIBN)* as a copolymerization initiator, produced by Sigma-Aldrich (USA), was purified by recrystallization in ethanol.

*NaCl of* “p.a.” grade was used without additional purification. Distilled water was used for preparation of solutions.

*Polyacrylic acid* with molecular weights of 2 × 10^3^ and 4.5 × 10^5^ from Sigma-Aldrich (USA) was used without further purification.

Purification of *organic solvents* (ethanol, ethyl ether of acetic acid, hexane) was carried out according to standard methods.

*Linear copolymers based on NVP and MA* were synthesized by substance initiation of monomer solutions in alcoholic solution in the presence of initiators hydrogen peroxide, ammonium persulfate and AIBN (5 × 10^−3^ M). The synthesis was carried out in sealed molybdenum glass ampoules in alcohol solution at 60 °C. The contents of the ampoules were purged with argon for 10–15 min to free the reaction mixture from oxygen. The polymer was isolated by resuspension from ethanol in an ethyl acetate/hexane mixture (1:1); after isolation, the polymer was dried in a vacuum cabinet to a constant weight.

## 3. Methods

The compositions of NVP-MA copolymers were determined by elemental analysis by determining the amount of nitrogen using Flash 2000 CHNS/O Elemental Analyzer equipment (Thermo Fisher Scientific Inc., USA).

*The pH of the polymer solutions and their blends* was adjusted with small amounts of 0.2 mol/L hydrochloric acid or sodium hydroxide and measured at a constant temperature of 25 °C on an “Ion Meter 3345” digital ionometer (Jenway Ltd., UK) with an accuracy of ±0.01 pH units.

*The optical density of* polymer solutions and their polycomplexes was measured on a UV spectrophotometer “UV-2401 PC” (Shimadzu, Japan) at λ = 400 nm (25 °C).

*Luminescence spectra of* PVP solutions and occurrence of NVP-MA copolymers in the presence of pyrene phosphor were recorded using an “FP-6200” spectrofluorometer (Jasco, Heckmondwike, UK). The excitation light wavelength was 335 nm. The values of I/I_13_ were calculated based on the intensities of the first vibration peak (I_1_) to the third (I_3_) at wavelengths of 383 and 373 nm, respectively.

*Diluted aqueous solutions of pyrene* (2 μM) were prepared according to the procedure used in [21,36]. Pyrene was dissolved in ethanol (0.4 mg/mL), and 100 μL of the obtained solution was transferred into a measuring flask (100 mL) and dried in a nitrogen current. The vessel was then filled with water and stirred continuously for 1 day. The solvent for the polymers and their complexes was the obtained pyrene solution.

*The weight-average molecular weights and molecular weight characteristics* of NVP-MA copolymers of different compositions were determined by gel permeation chromatography using the ALC 201 GPC instrument (Waters, UK). Tetrahydrofuran was used as the eluent (mobile phase) at a flow rate of 1.0 mL/min. Molecular masses were calibrated according to polystyrene standards.

*The viscosity of the copolymer aqueous solutions* was measured using an Ubbelode viscometer at 20 °C with an accuracy of ±0.1 °C. The characteristic viscosity [η] was calculated graphically from the relationship η_red.-*C*_, where η_red_ is the reduced viscosity of a solution of a given concentration *C*, by extrapolating a straight line to the intersection with the ordinate axis. The area cut-off on the y-axis corresponded to η.

All characteristic measurements were carried out after reaching the equilibrium state, and control measurements were performed to prove that the state of the system had ceased to change.

## 4. Results

Table 1 shows the characteristics of the NVP-MA copolymers obtained at different compositions of the initial monomer mixture (IMM). It can be seen that, due to the higher activity of MA in radical copolymerization (compared to the activity of NVP), the composition of the copolymers is enriched with MA content compared to its concentration in the IMC. At the same time, as can be seen from the data in Table 1, the M_W_ of the copolymers formed in the process of copolymerization increases with increasing concentrations of MA in IMM. This is also due to the increase in the growth rate of the macromolecular chain with increasing concentrations of the more active MA monomer in IMM.

It can also be seen that the value η is not directly related to the copolymer M_W_. It is known [reference to a textbook on physical chemistry] that the characteristic viscosity η depends not only on the M_W_ of copolymers, but also on their conformation in solution. The larger the volume occupied by a macroblob in solution, the higher its characteristic viscosity. It is obvious that as the hydrophobic comonomer content in the macromolecular composition increases, the thermodynamic quality of the solvent (water) deteriorates, leading to the formation of more compact macromolecular clusters.

Therefore, as can be seen from Table 1, copolymers with a high MA content in their composition (49.0 and 55.9 mol.%) have lower values of η in aqueous solutions, despite the fact that they are characterised by higher M_W_ values compared to copolymers with lower concentrations of MA in their macromolecular composition (47.1 mol.% and lower).

IR spectra illustrating the behavior of the systems considered are shown in the Supplementary Materials, Figures S1 and S2.

**Figure 1:**
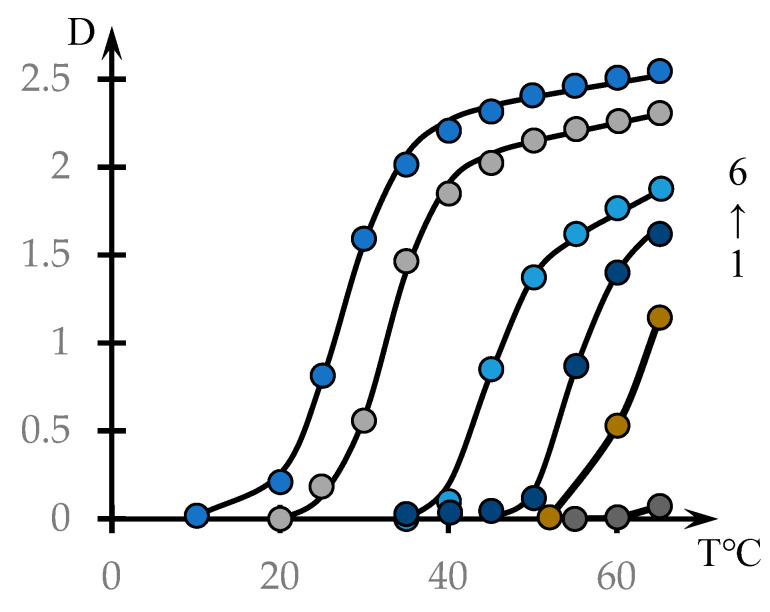
Effect of temperature on the optical density of aqueous solutions of NVP-MA copolymers: [NVP]:[MA] = 71.0:29.0 (1); 60.0:40.0 (2); 52.9:47.1 (3); 51.0:49.0 (4); 44.1:55.9 (5); 39.9:60.1 mol.% (6). C = 0.1 mol.%.

Figure 1 shows the dependences of the optical density of NVP-MA CPL solutions on their temperature (points). The solid lines were drawn using the phenomenological theory, which is discussed in Discussion Section.

As expected, for copolymers with a high content of hydrophobic MA links, the phase transition occurs at lower temperatures. It should also be noted that in solutions of the investigated concentrations, the NVP-MA copolymers with a 71.0:29.0 mol.% composition do not form a precipitate in the whole temperature range investigated.

**Figure 2:**
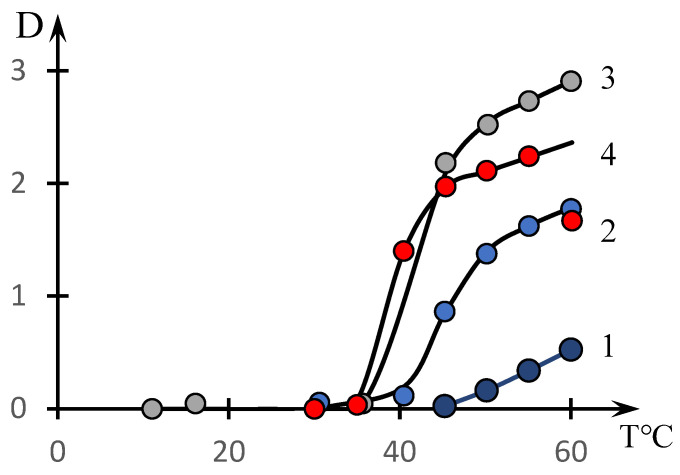
Effect of temperature on the optical density of aqueous solutions of NVP-MA copolymers: _Copolymer_ = 0.01(1); 0.1 (2); 0.5 (3); 1 mol.% (4). [NVP]:[MA] = 51.0:49.0 (4) mol.%.

Figure 2 shows similar dependences for different concentrations of the copolymer with the composition [NVP]:[MA] = 49.0:51.0 mol.% (dots). The solid lines in this figure show the approximations constructed using the formula discussed in the next section.

It can be seen that the phase transition temperature decreases significantly with increasing solution concentration.

Figure S3 in the Supplementary Materials, Point 2, shows the same dependences as in Figure 2, but for the composition [NVP]:[MA] = 44.1:55.9. It can be seen that a significant change in optical density only occurs in more concentrated solutions and at sufficiently high temperatures. Further reduction in the hydrophobic component (up to the composition NVP-MA 71.0:29.0 mol.%) and above results in the solution remaining transparent over the whole temperature range investigated.

**Figure 3:**
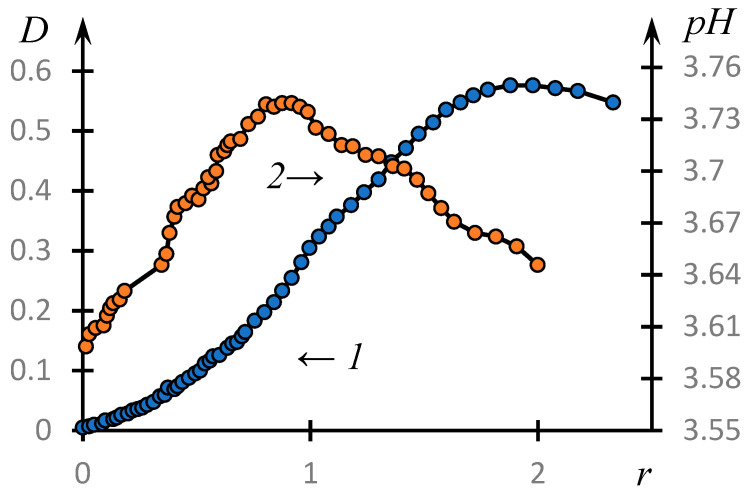
Turbidimetric (1) and potentiometric titration curves (2) of PAA solutions with NVP-MA solutions: [NVP]:[MA] = 51.0:49.0 mol.%; [CPL] = [PAA] = 0.01 M; MM (PAA)= 450,000.

Figure 3 shows the curves of the turbidimetric (1) and potentiometric titration (2) of PAA solutions with NVP-MA solutions: $r=\frac{\left[NVP-MA\right]}{\left[PAA\right]}$.

**Figure 4:**
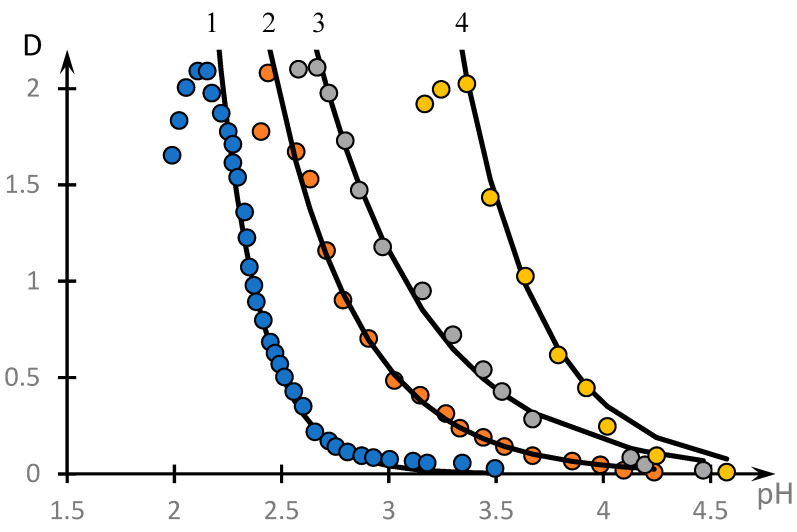
Effect of low-molecular-weight salt on the formation of NVP-MA copolymer complex with PAA: C_NaCl_ = 0 (1), 0.005 (2), 0.01 (3), 0.1 (4); [NVP]:[MA] = 52.9:47.1 mol.%; MM (PAA) = 250,000; [NVP-MA] = [PAA] = 0.01 M.

It can be seen that the formation of a complex does take place in the system under consideration. The optical density reaches its maximum value at a concentration ratio approximately equal to 1.8. The influence of low-molecular-weight salt on the complex formation is illustrated by Figure 4, which shows the temperature dependences of the optical density of the solution at different values of the concentration of low-molecular-weight salt *C* (points). The solid lines represent the curves obtained using the approximation discussed in the next section. It can be seen that with increasing concentration of low-molecular-weight salt in the solution, the obtained curves shift towards higher pH values.

In accordance with the methodology used, for example, in [36,51], to study the hydrophobic–hydrophilic balance, the luminescence emission spectra of pyrene, which serves as a marker additive in the solutions of the polymers under study, are used. The luminescence spectrum of pyrene is rather complex; however, in it, one can confidently distinguish the first ($\lambda_{1}$ = 373.0 nm) and the third vibrational peaks ($\lambda_{3}$ = 383.5 nm). The ratio of the amplitudes of these peaks $\frac{I_{3}}{I_{1}}$ according to the methodology used in [36,51] is considered to be a measure of the degree of hydrophobicity of the medium. The higher this ratio, the more pronounced the hydrophobic interactions in the solution under study.

**Figure 5:**
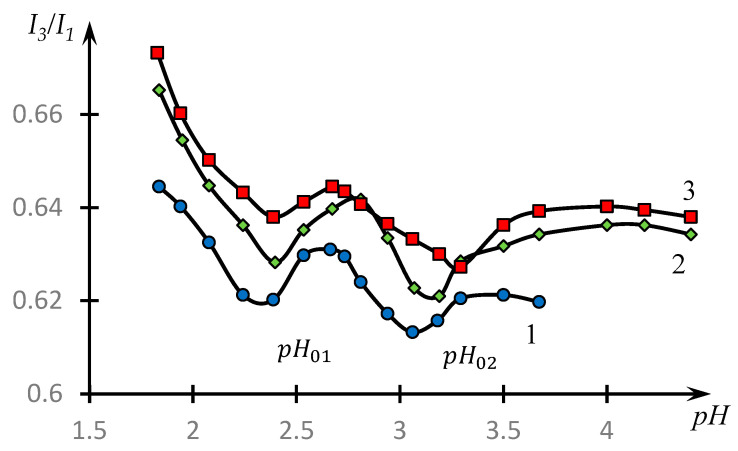
Dependence of *I*_3_*/I*_1_ on the pH of the medium for an aqueous solution of pyrene in the presence of an equimolar mixture of NVP-MA/PAA CPLs for CPLs of different compositions: [NVP]:[MA] = 71.0:29.0 (1); 52.9:47.1 (2); and 44.1:55.9 (3) mol.%. MM (PAA) = 250,000; NVP-MA CPL = [PAA] = 0.01 M.

Figure 5 shows the dependences of the ratio $\frac{I_{3}}{I_{1}}$ on the pH of the medium for the aqueous solution of pyrene in the presence of an equimolar mixture of NVP-MA and the PAA copolymer for copolymers of different compositions.

It can be seen that the obtained dependences have two pronounced minima. Looking ahead, we note that in [51], this fact was interpreted through the conclusion about the existence of HIAs, the nature of which was subsequently revealed in [45,52].

## 5. Discussion

Figure 1 shows that the character of the dependence of optical density on temperature for the studied solutions qualitatively differs from similar curves obtained in [46] when studying solutions of the copolymers N-Vinylpyrrolidone and Vinyl Propyl Ether, as well as in [53] when studying the formation of complexes between nonionic polymers and polyacid.

Namely, the temperature dependences of the optical density of the solution obtained in these works are described by the following formula:

(1) $$D=D_{0}\frac{exp\left[(T-T_{0})/\tau \right]}{1+exp\left[(T-T_{0})/\tau \right]}$$

where $D_{0}$ is the amplitude of optical density changes, $T_{0}$ is the phase transition temperature, and τ is a constant characterizing the steepness of the phase transition, which has the dimension of temperature.

We emphasize that the phase portrait method has been proposed in our works and is still mainly used by our research group [46,53]. In this and our previous work, it is shown that our proposed method allows us to obtain non-trivial information about the nature of phase transitions. This work provides further confirmation of the feasibility of this method in polymer science and an argument for its wider use.

Curve (1) describes the transient process between two constant limit values equal to 0 and $D_{0}$, respectively.

The curves presented in Figure 1 and Figure 2 do not meet this criterion. As the temperature increases, they do not reach a plateau, as is characteristic of the curves studied in the cited works [46,53].

More precisely, these curves admit approximation by dependences of the following form:

(2) $$D=\left(\beta T+D_{0}\right)\frac{exp\left[(T-T_{0})/\tau \right]}{1+exp\left[(T-T_{0})/\tau \right]}$$

where β  is a constant.

This is the approximation used in the construction of solid curves (3) through (6) in Figure 1 and curves (3) through (5) in Figure 2.

In fact, Formula (2) means that the temperature dependence of the optical density can be decomposed into two factors. The first of them is linear, and the second one describes a transient process of the same type as the one studied in [46,53].

**Figure 6:**
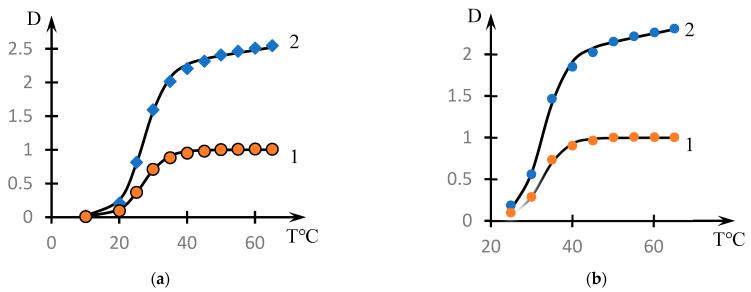
Illustration of the decomposition of the curves of Figure 1 into multipliers by Formula (2): decomposition of (**a**) curve 6 and (**b**) curve 5. Curves 1 correspond to the approximation by Formula (1), curves 2 correspond to the approximation by Formula (2).

The representation of form (2) is illustrated by Figure 6. Curve 1 in these figures corresponds to the coefficient describing the transient process, and curve 2 to the approximation using Formula (2).

The parameters included in the dependences of form (2), on which the solid curves presented in Figure 1 are plotted, are shown in Table 2.

It is noteworthy that the parameter *τ*, which characterizes the steepness of the phase transition, decreases significantly with decreasing hydrophobic component content, i.e., the phase transition becomes more abrupt.

**Figure 7:**
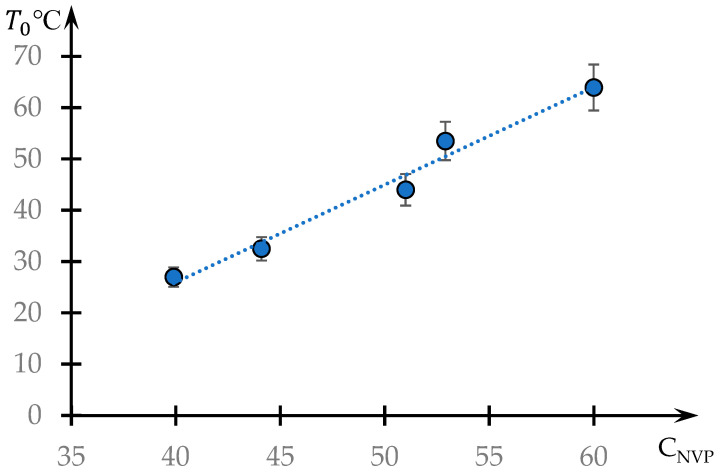
Dependence of the phase transition temperature $T_{0}$ on the content of the hydrophobic component C_NVP_.

The dependence of the phase transition temperature on the NVP content is shown in Figure 7. This dependence is close to linear.

Taking into account the obtained results, as well as the results of [45,53] and the considerations expressed therein, the fact that the considered curves are approximated by Formula (2) can be interpreted as follows.

An isolated macromolecular tangle, which contains both hydrophilic and hydrophobic links, does not necessarily have to undergo a phase transition, i.e., to jump from one state to another. Consequently, for a dilute solution (i.e., under conditions where the interaction between macromolecules is minimized), the temperature dependence of the optical density can be close enough to linear.

For macromolecules of this type, the phase transition can be predominantly associated with the formation of HHAs, the existence of which was demonstrated in [46].

Such associates, as well as the HIAs studied in [45,52], are polymer meshes existing in a dynamic regime. The bonds between the elements of the mesh are constantly broken and formed again. Associates of the above types occupy an intermediate position between true solutions, hydrogels and interpolymer complexes. They differ from hydrogels in that the mesh is not stable, and hence, the system has fluidity. They differ from interpolymer complexes in that the mesh is very branched, resembling hydrogels in structure (with the difference that the bonds forming it are not stable). Note also that, unlike classical IPCs, it does not make sense to talk about the size of HHAs. These associates are unstable meshes that exist in the dynamic regime. Accordingly, such networks cover the entire volume of the solution.

If the formation of HHAs is included, the observed behavior of the studied solutions described by Formula (2) can be interpreted as follows.

Provided that the isolated macromolecules of the studied polymer do not experience a jump-like phase transition, the temperature dependence of the optical density should remain hollow, which is reflected by the first coefficient in Formula (2). The sharp phase transition, as well as the change in optical density close to a jump-like one, is due to the fact that with increasing temperature, the degree of swelling of the tubules decreases, as hydrophobic interactions in them increase. This leads to the destruction of the dynamic mesh-HHAs. Upon completion of such a transition, the temperature dependence of optical density becomes close to linear, i.e., it corresponds to the behavior of isolated macromolecules in which hydrophobic interactions increase.

**Figure 8:**
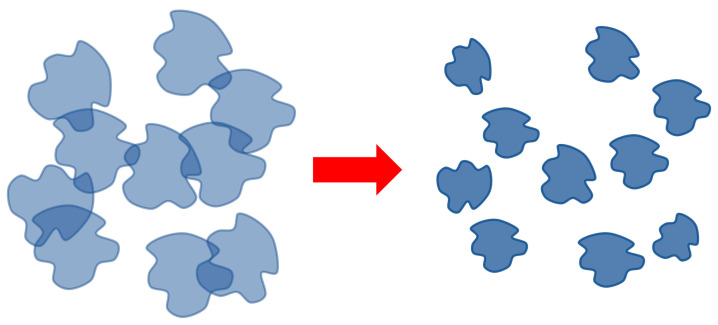
Scheme of HHA breakdown with increasing temperature/increasing hydrophobic interactions.

Schematically, the disintegration of the dynamic mesh-HHAs, proceeding due to a decrease in the degree of swelling of the tubules, is illustrated in Figure 8.

The linear character of the phase transition temperature’s dependence on the concentration of the hydrophobic component in the studied copolymer (Figure 7) also agrees with the proposed interpretation. If the phase transition is caused by changes in the swelling degree of a single ball, which depends linearly (or close to it) on the temperature, then the temperature of the dynamic mesh destruction should also depend linearly on the content of the hydrophobic component in the ball.

The behavior of the solution of the studied copolymer at changing concentrations (Figure 2) also agrees with the proposed interpretation. The curves presented in Figure 2 are also approximated by dependence (2). The exception is curve 1, which corresponds only to the initial stage of the phase transition.

**Figure 9:**
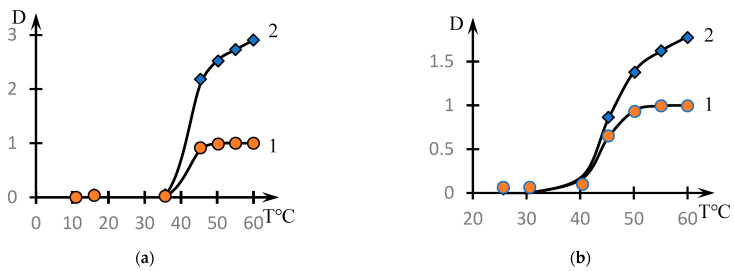
Examples of decomposition of curves of Figure 2 into multipliers by Formula (2): decomposition of (**a**) curve 2 and (**b**) curve 3. Curves 1 correspond to the approximation by Formula (1), curves 2 correspond to the approximation by Formula (2).

Examples illustrating the decomposition of the curves shown in Figure 2 by the factors are presented in Figure 9. The values of the approximation parameters are shown in Table 3.

It can be seen that the phase transition temperature corresponding to the second coefficient in Formula (2) decreases markedly with increasing copolymer concentration in the solution. We also emphasize that with increasing concentration of the solution, the phase transition becomes sharper, which corresponds to the decrease in the parameter τ.

These facts are explained by the fact that an increase in the solution concentration leads to the formation of a more branched dynamic network-HHA, which, with increasing temperature, first disintegrates into separate fragments, and then, into isolated macromolecular clubs.

**Figure 10:**
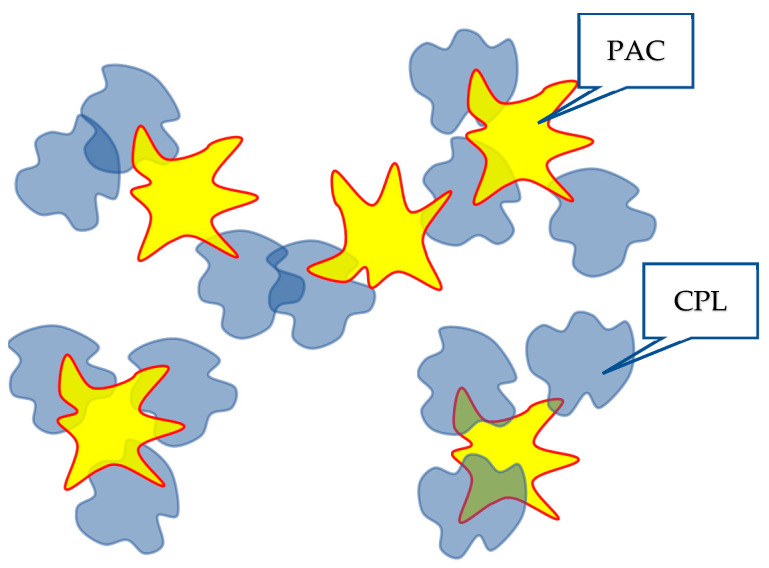
Illustration of the influence of HHAs on the character of the interaction of the studied copolymers with PAA.

It is of interest to understand how the formation of HHAs between macromolecules of the same type can influence the formation of interpolymer complexes. As noted above, it was previously shown [45] that along with classical interpolymer complexes, HIAs can also be formed in solutions containing interacting macromolecules. The mechanisms of the formation of associates of both mentioned types are similar. Consequently, there is every reason to assume that the formation of a dynamic mesh formed in a solution containing a copolymer of the type under consideration and PAA will be influenced by the bonds formed between clubs of both the same and different types. Schematically, the formation of a grid of the corresponding structure is illustrated in Figure 10.

The nature of the connections between the meshes of different natures is illustrated by Figure S4, which is included in the Supplementary Materials Point 3.

It should also be emphasized that although the networks studied are formed in a dynamic regime, they belong to a system in thermodynamic equilibrium.

The formation of this kind of dynamic mesh cannot but depend on the ratio of concentrations of the copolymer and PAA under study $r=\frac{\left[NVP-MA\right]}{\left[PAA\right]}$. Indeed, at r≪1, one should expect the formation of HIAs in accordance with the mechanism established in [45]. In this case, the copolymer tubules serve as a kind of cross-agent connecting PAA molecules into an unstable dynamic mesh. At r≈1, one can expect the formation of structures close to classical IPC. Finally, at r≪1, it is acceptable to expect that in the solution, an HHA close to that formed in the solution containing only the copolymer under study will be formed. Between these limiting cases, obviously, there are transition regions.

The above assumptions are supported by experimental data.

**Figure 11:**
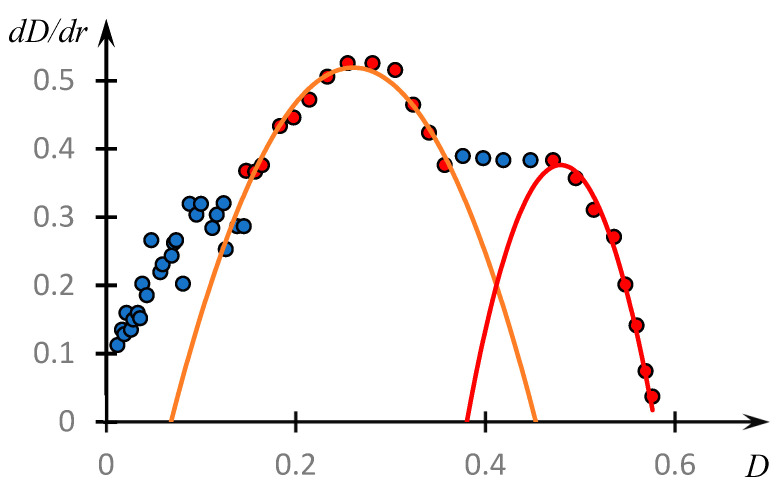
Phase portrait of the dependence of optical density on the ratio *r* of copolymer and PAA concentrations (Figure 3). The points coloured in different colours correspond to different parts of the same phase portrait: red shows the parts approximated with good accuracy by parabolic curves.

Figure 11 shows the phase portrait of the dependence of the optical density on the parameter *r*, i.e., the dependence of the derivative $\frac{dD}{dr}$ obtained by numerical differentiation of the experimental data from D.

It can be seen that on the obtained curve, we can distinguish areas that are approximated by parabolas with high accuracy.

These parabolas are expressed by the following formulas, the numerical values of the coefficients in which are obtained by the least squares method.

(3) $$\frac{dD}{dpH}=-14.0D^{2}+7.3D-0.43$$

(4) $$\frac{dD}{dpH}=-38.3D^{2}+36.7D-8.43$$

Curves (3) and (4) reach a maximum at D=0.26 and D=0.48, respectively. These values correspond to the values r=0.92 and r=1.42.

We emphasize that the value r=0.92,  which corresponds to the maximum of the first parabola under consideration, also corresponds to the maximum of the dependence pH(r), curve 2, Figure 3. This allows us to assume that the composition of the unstable dynamic grid of the hydrophilic association corresponds to this ratio of the concentrations of the reacting components. The formation of classical IPC proceeds under the condition that the copolymer concentration is about 1.4 times higher than the PAC concentration (the second of the parabolic plots). Apparently, this is connected with the fact that macromolecular clubs containing hydrophobic components interact quite effectively not only with polyacid clubs, but also with each other (Figure 10).

This conclusion correlates with the results presented in Figure 5. The presence of two minima of dependences $\frac{I_{3}}{I_{1}}(pH)$, in accordance with the results of [25,36], indicates that along with classical PKI in the system under consideration, HIAs are also formed. The most effective COE formation takes place in the area corresponding to $pH_{02}$ and HIA formation in the area corresponding to $pH_{01}$.

The existence of objects of different natures, which can be formed in the considered system in parallel, among other things, means that the question about the values of critical parameters (pH, concentration of low-molecular salt, etc.) at which the phase transition occurs is not trivial.

The fact that a change in the acidity of the medium does induce the phase transition is directly evidenced by the curves presented in Figure 4; however, the question arises as to what should be taken as a critical value of pH.

**Figure 12:**
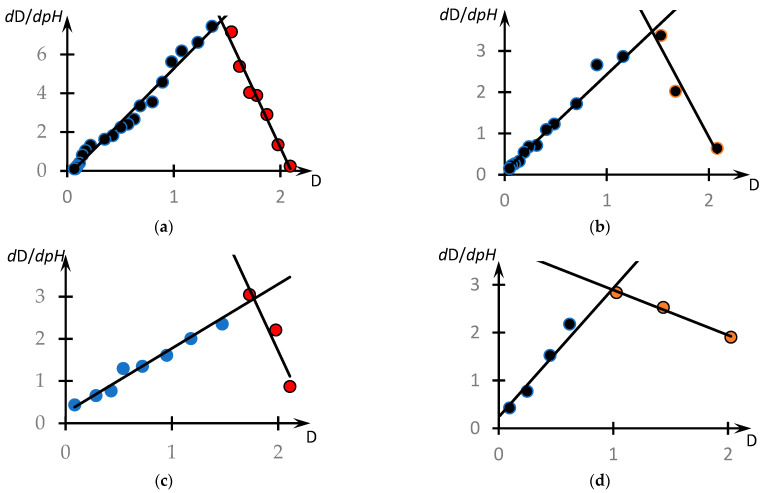
Phase portraits of the curves presented in Figure 4. Curves 1–4 from Figure 4 correspond to (**a**–**d**), different colours highlight different points of the same phase portrait, but belonging to sections approximated by different straight lines.

This question can be answered by analysing the phase portraits of the curves presented in Figure 4. They are shown in Figure 12.

These curves are also plotted according to the methodology used in [46,53]. Specifically, these figures show the dependence of the derivative $\frac{dD}{dpH}$ taken with the opposite sign from D, obtained on the basis of experimental data. The values of the derivatives were calculated according to the standard technique through the approximation of a separate section of the experimental curve using straight lines and determination of the tangent of the angle of its slope. In the case of Figure 12a, the local straight line was drawn at seven points, and for the other curves, at five points.

It can be seen that, in contrast to the results obtained in the cited works [46,53], the phase portraits obtained differ significantly from the parabolic ones. All the phase portraits obtained break up into two rectilinear sections, i.e., the range of *pH* change breaks up into at least two subranges corresponding to the different natures of the dependence of optical density on the acidity of the medium.

The linear character of the phase portrait fragments means that in the corresponding ranges of pH change, the dependence D(pH) obeys the following first-order differential equations.

(5) $$\frac{dD}{dpH}=-k_{1}D$$

(6) $$\frac{dD}{dpH}=k_{2}D-k_{3}$$

Consequently, the curves presented in Figure 4 should be approximated with high accuracy by segments of exponential curves. Indeed, the solutions of Equations (3) and (4) have the following form:

(7) $$D=D_{0}\;exp\left(-k_{1}pH\right)$$

(8) $$D=\frac{k_{3}}{k_{2}}-D_{0}\;exp\left(k_{2}pH\right)$$

**Figure 13:**
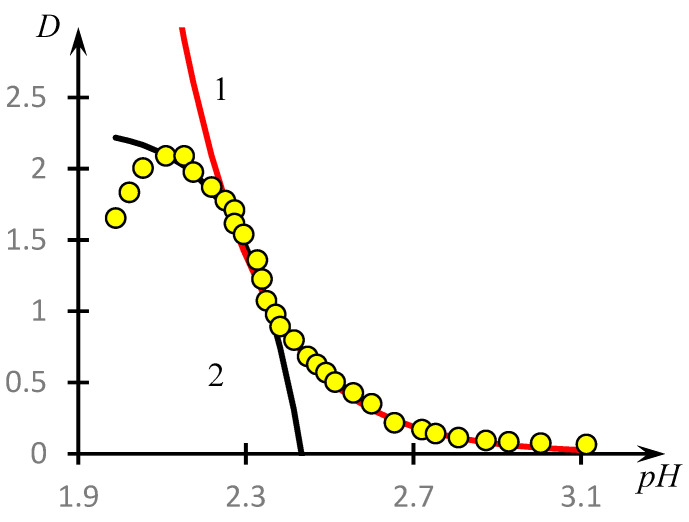
Approximation of curve 1, Figure 4, by two segments of exponential curves—solutions of Equations (5) and (6); curve 1—solution of Equation (5), curve 2—solution of Equation (6), yellow circles—experimental data.

The corresponding construction for curve 1, Figure 4, is shown in Figure 13. It can be seen that the curve under consideration is indeed approximated with high accuracy by the solutions of Equations (7) and (8).

It is acceptable to assume that different areas on the considered curve also correspond to interpolymer objects of different characters. Indeed, with increasing pH, the degree of ionization of PAC increases, which is obviously accompanied by an increase in the size of the clubs. Consequently, at relatively small pH, tubules of the copolymer under consideration can serve as a kind of cross-agent inducing the formation of bonds between PAA tubules, which ensures the formation of HIAs. On the contrary, at large values of pH, this possibility is lost.

This creates certain difficulties in determining the numerical value of pH at which the phase transition occurs (in any case, on the basis of such curves as shown in Figure 4).

Indeed, if different parts of such curves belong to objects of different natures, they may respond to changes in other control parameters (e.g., the concentration of low-molecular-weight salt in solution) in different ways. Moreover, as follows from the results, the addition of a low-molecular-weight salt to the PAA solution causes ion exchange effects, as a result of which the distribution of low-molecular-weight ions over the solution volume is obviously not homogeneous, which may affect the nature of the formation of such branched structures as HIAs.

For the system under consideration, this difficulty can be overcome as follows.

Solution (7) can be represented in the following form:

(9) $$D=exp\left[-k_{1}\left(pH-pH_{03}\right)\right]$$

where

(10) $$pH_{03}=\frac{lnD_{0}}{k_{1}}$$

Proceeding from (9), it is reasonable to treat value (10) as a critical value of pH at which there is a phase transition associated with the formation of classical IPC. It should be emphasized that the fragment of the declining exponential curve is known to correspond to the formation of the IPC. The corresponding numerical values of $pH_{03}\;$ obtained using the curves of Figure 4 are shown in Table 4. It can be seen that $pH_{03}$ monotonically increases with increasing concentrations of sodium chloride in the solution.

## 6. Conclusions

The results of this work show that the nature of phase transitions in solutions of thermosensitive copolymers containing hydrophobic functional groups is determined by at least two factors—the change in the state of macromolecular clubs, in which hydrophobic interactions are strengthened or weakened, as well as the formation of unstable meshes (HHAs). Such associations exist in a dynamic mode, i.e., the bonds between macromolecular clubs are continuously broken and reappear. It is essential that the change in the state of an isolated macromolecular tangle need not be discontinuous. A sufficiently sharp phase transition in this case is determined by the destruction of the HHAs.

The factors leading to the formation of HHAs also affect the nature of the interaction of copolymers of the considered type with PAA. In this case, depending on the conditions, a rather wide range of different products of interpolymer interactions can be formed. In addition to classical IPCs, HIA, also representing dynamic networks, can be formed in such interactions, and the character of their formation can be influenced by the bonds between identical macromolecular bundles containing hydrophilic groups.

## Figures and Tables

**Table 1:** Composition of copolymers and some of their properties in aqueous solutions.

Composition of IMM, mol.% [NVP]:[MA]	Compositions of Copolymers, mol.% [NVP]:[MA]	[η], dL/g	M_W′_ (kDa)	M_N′_ (kDa)
90:10	71.0:29.0	0.43 ± 0.5	60.6 ± 0.5	36.7 ± 0.5
80:20	60.0:40.0	0.47 ± 0.5	73.8 ± 0.5	45.8 ± 0.5
70:30	52.9:47.1	0.51 ± 0.5	89.7 ± 0.5	53.3 ± 0.5
60:40	51.0:49.0	0.41 ± 0.5	101.4 ± 0.5	57.4 ± 0.5
50:50	44.1:55.9	0.23 ± 0.5	132.1 ± 0.5	69.2 ± 0.5

**Table 2:** Parameters of approximation of the curves presented in Figure 1 by Formula (2).

C_NVP_	β	$D_{0}$	τ
39.9 ± 0.2	0.008 ± 0.005	2.01 ± 0.05	3.5 ± 0.2
44.1 ± 0.2	0.010 ± 0.005	1.65 ± 0.05	2.9 ± 0.2
51 ± 0.2	0.025 ± 0.005	0.22 ± 0.05	2.4 ± 0.2
52.9 ± 0.2	0.044 ± 0.005	−1.19 ± 0.05	2.0 ± 0.2

**Table 3:** Parameters of approximation of the dependences presented in Figure 2 using Formula (2).

C_pol_, mol.%	$D_{0}$	β	$T_{0}$	τ
0.1	−0.08 ± 0.3	0.031 ± 0.005	44.0 ± 0.5	2.2 ± 0.2
0.5	0.77 ± 0.3	0.036 ± 0.005	42.0 ± 0.5	1.7 ± 0.2
1	0.80 ± 0.3	0.026 ± 0.005	39.0 ± 0.5	1.3 ± 0.2

**Table 4:** Dependence of one of the critical pH values on the concentration of sodium chloride.

*C_NaCl_*	0	0.005	0.01	0.1
$pH_{03}$	2.37 ± 0.3	2.76 ± 0.3	3.07 ± 0.3	3.63 ± 0.3

## Data Availability

The authors confirm that the data supporting the findings of this study are available within the Supplementary Materials.

## Supplementary Materials

The following supporting information can be downloaded at: https://www.mdpi.com/article/10.3390/polym16050584/s1, Figure S1: IR spectra of NVP (1), PAK (2) and NVP-MA copolymer 44.1:55.9 mol% (3); Figure S2: IR spectra of NVP-MA-PAA polycomplex films at their ratios of 1:4 (1), 1:2 (2), 1:1 (3) and 3:2 mol% (4) in the blend; [NVP]:[MA] = 44.1:55.9 mol%; Figure S3: Effect of temperature on the optical density of aqueous solutions of NVP-MA copolymers [NVP]:[MA] = 60.0:40.0; Cpolymer=0.001 (1); 0.01 (2); 0.1 (3); 0.5 (4); 1 mol% (5); Figure S4: Schematic of connections between studied macromolecular clubs in aqueous solution.
