# Supplementary material for: Effect of the Formation of Hydrophilic and Hydrophobic–Hydrophilic Associates on the Behavior of Copolymers of N-Vinylpyrrolidone with Methyl Acrylate in Aqueous Solutions
Source: Polymers (Basel). 2024 Feb 21;16(5):584. doi: 10.3390/polym16050584 (PMC10934458; doi:10.3390/polym16050584)
Supplement: Supplementary file 1 [file polymers-16-00584-s001.zip › polymers-2833952-supplementary.pdf]

Point 1

In this work, IR spectra were obtained by casting on polyethylene substrates. Figure S1 shows the spectra of NVP, PAA and NVP-MA copolymer (44.1:55.9 mol%). Figure S2 shows the mixtures of copolymer with PAK at different ratios at pH = 3.6. It can be seen that the IR spectra of the copolymer-PAA blends undergo significant changes with increasing copolymer content in their composition. The absorption band at 1659 cm^{-1} of the vibrations of the carbonyl group of the lactam ring of NVP shifts to the region of smaller wave numbers up to the ratio [NVP-MA]/[PAA]=1:1 mol (1639 cm^{-1}), with further increase of the copolymer content a rather strong increase of the absorption intensity of the unbound carbonyl group of NVP (wave number 1653 cm^{-1}) is observed and a characteristic shoulder at 1600 cm^{-1} of the vibrations of the complex-bound group C=O appears.

The band responsible for the valence vibrations of the PAA carbonyl group (1715 cm^{-1}) also undergoes a change in position, i.e. with increasing copolymer content in the mixture, it is shifted to the region of high wave numbers (up to 1728 cm^{-1}), probably associated with the destruction of strong dimeric groups of PAA and the formation of intermolecular hydrogen bonds with the copolymer.

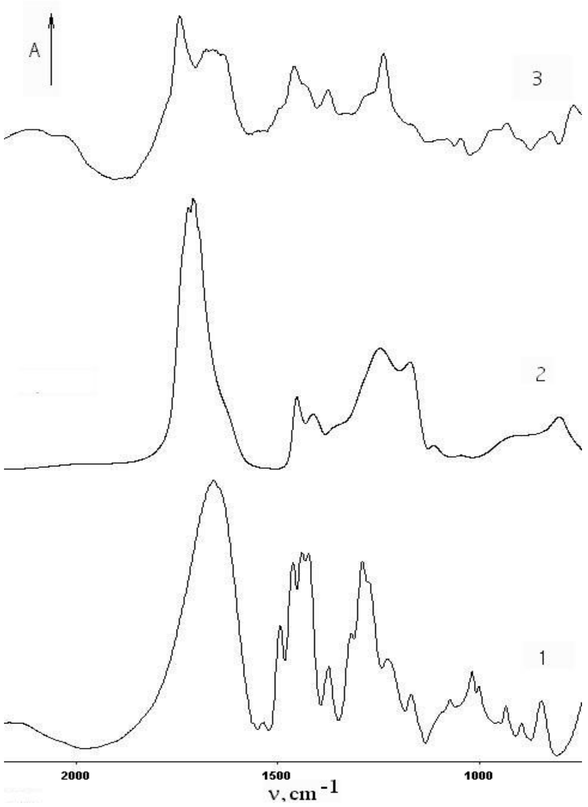

Figure S1- IR spectra of NVP (1), PAK (2) and NVP-MA copolymer 44.1:55.9 mol% (3)

A series of absorption bands at 1268, 1238, 1192, 1172, 1148 cm^{-1} of MA ester groups in copolymer/PAA blends are associated with intramolecular hydrophobic interaction of individual MA links in the copolymer. In comparison, the IR spectra of the copolymer show no such splitting of the ester band.

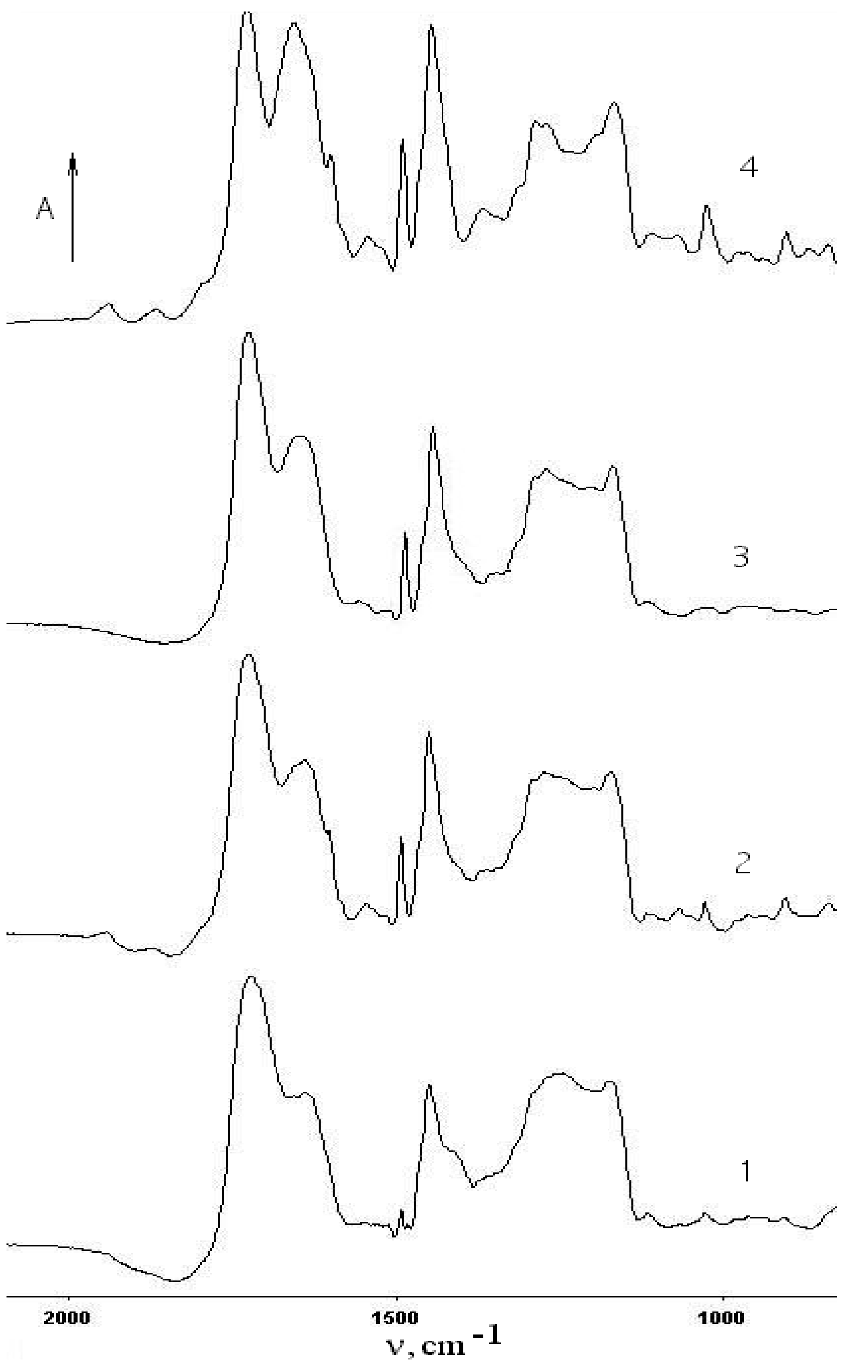

Figure S2 - IR spectra of NVP-MA-PAA polycomplex films at their ratios of 1:4 (1), 1:2 (2), 1:1 (3) and 3:2 mol% (4) in the blend; [NVP]:[MA] = 44.1:55.9 mol%;

Point 2

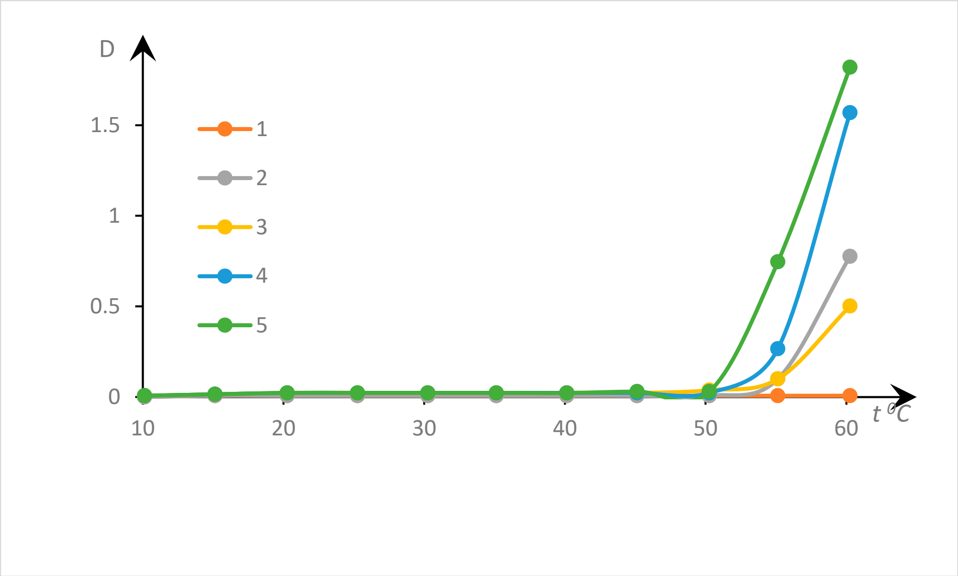

Figure S3 - Effect of temperature on the optical density of aqueous solutions of NVP-MA copolymers [NVP]:[MA] = 60.0:40.0; $C_{polymer}$=0.001 (1); 0.01 (2); 0.1 (3); 0.5 (4); 1 mol% (5)

Point 3

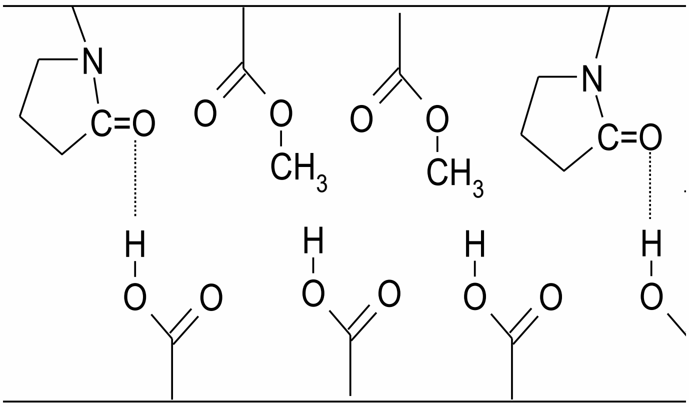

Figure S4 - Schematic of connections between studied macromolecular clubs in aqueous solution

Point 4

The most interesting prospects for the practical application of the results obtained are related to the improvement of aromatherapy methods, in general, for the aromatisation of premises. This market is very developed, and the cost of products presented on it (aromatic candles and their analogues) in Kazakhstan reaches several tens of US dollars. Providing controlled release of aromatic components from the solution will allow, among other things, to realise their electronic analogues. The next step in this direction is the controlled separation of the mixture of aromatic components (up to the creation of devices providing conjugation of variations of the created aroma to the beat of music, etc.).
